# Increased hospitalizations for decompensated heart failure and acute myocardial infarction during mild winters: A seven-year experience in the public health system of the largest city in Latin America

**DOI:** 10.1371/journal.pone.0190733

**Published:** 2018-01-04

**Authors:** Renato Kawahisa Levin, Marcelo Katz, Paulo H. N. Saldiva, Adriano Caixeta, Marcelo Franken, Carolina Pereira, Salo V. Coslovsky, Antonio E. Pesaro

**Affiliations:** 1 Hospital Israelita Albert Einstein, Sao Paulo, Sao Paulo, Brazil; 2 Instituto de Estudos Avançados da Universidade de São Paulo, Sao Paulo, Sao Paulo, Brazil; 3 Robert F. Wagner School of Public Service, New York University, New York, New York, United States of America; Instituto de Cardiologia J F Cabral, ARGENTINA

## Abstract

**Background:**

In high-income temperate countries, the number of hospitalizations for heart failure (HF) and acute myocardial infarction (AMI) increases during the winter. This finding has not been fully investigated in low- and middle-income countries with tropical and subtropical climates. We investigated the seasonality of hospitalizations for HF and AMI in Sao Paulo (Brazil), the largest city in Latin America.

**Methods:**

This was a retrospective study using data for 76,474 hospitalizations for HF and 54,561 hospitalizations for AMI obtained from public hospitals, from January 2008 to April 2015. The average number of hospitalizations for HF and AMI per month during winter was compared to each of the other seasons. The autoregressive integrated moving average (ARIMA) model was used to test the association between temperature and hospitalization rates.

**Findings:**

The highest average number of hospital admissions for HF and AMI per month occurred during winter, with an increase of up to 30% for HF and 16% for AMI when compared to summer, the season with lowest figures for both diseases (respectively, HF: 996 *vs*. 767 per month, p<0.001; and AMI: 678 *vs*. 586 per month, p<0.001). Monthly average temperatures were moderately lower during winter than other seasons and they were not associated with hospitalizations for HF and AMI.

**Interpretation:**

The winter season was associated with a greater number of hospitalizations for both HF and AMI. This increase was not associated with seasonal oscillations in temperature, which were modest. Our study suggests that the prevention of cardiovascular disease decompensation should be emphasized during winter even in low to middle-income countries with tropical and subtropical climates.

## Introduction

Coronary artery disease and heart failure (HF) are leading causes of morbidity and mortality worldwide.[1] In low- and middle-income countries, an aging population has been associated with an increased prevalence of both diseases. In Brazil, HF has become a leading cause of hospitalization while acute myocardial infarction (AMI) has become the main cause of death.[2, 3]

High-income countries with temperate climates are characterized by large seasonal oscillations in ambient temperature. In these countries, the association between winter and decompensated cardiovascular diseases has been clearly demonstrated. [4, 5] Biological mechanisms linking low temperatures to higher cardiovascular risk include persistently higher sympathetic nervous system activation, uncontrolled hypertension, and an increased incidence of respiratory diseases. Social and environmental mechanisms that hinge not only on low temperatures but on winter conditions more generally—such as shorter days, reduced physical activity, depression, and higher pollution levels—may also explain higher cardiovascular risk.[6, 7]

In low- and middle-income countries with a tropical and subtropical climate, the effect of mild winters on cardiovascular decompensation has not been fully investigated. In these countries, temperature does not oscillate much across seasons but other social and environmental factors such as precarious housing conditions, lack of thermal insulation, and greater pollution might increase the seasonal effect of winter on risk.[8–10]

In our study, we investigated the seasonality of hospitalizations for HF and AMI and tested the association between monthly average temperatures and hospitalizations for these diseases in the public health system of Sao Paulo, Brazil, the largest city in Latin America. We hypothesize that in Sao Paulo, the winter season is associated with increased cardiovascular risk even if seasonal temperature oscillations remain modest. We also speculate that this association might be rooted in the socio-economic circumstances faced by our studied population, i.e. the users of the public health system, such as greater exposure to air pollution, lack of thermal insulation, precarious housing, and other tribulations faced by those who live with low-income in such a megacity.

## Methods

This was an observational, retrospective study of data obtained from 61 public hospitals in Sao Paulo. [11] The data was recorded prospectively on a monthly basis from January 2008 to April 2015 by the National Registry of Public Health, which is maintained by the Brazilian Public Health System. [12] To record the data, the National Registry requires that hospitals submit an official form (“Hospital Admission Authorization”) for each patient that is admitted under the auspices of the Brazilian Public Health System. This form contains the primary admission diagnosis and other patient data.

For our study, the inclusion criteria were: Age >20 years and admission for HF or AMI as defined by the International Classification of Diseases (ICD-10) [13], in which code I50 represents HF and code I21 represents AMI. Data on monthly average temperatures were obtained from the Environmental Sanitation Technology Company of Sao Paulo, based on 24 hourly measurements per day, from six meteorological stations in the city.[14] To calculate monthly average temperatures for the city, we first calculated average temperatures for each station and month, and then calculated the mean temperature levels for all stations for each month. This study was conducted in accordance with The Code of Ethics of the World Medical Association (Declaration of Helsinki) for experiments involving humans and was approved by the Institutional Review Board of the Hospital Israelita Albert Einstein. The study was granted a waiver for informed consent.

## Statistical analysis

For each diagnosis, the average number of hospitalizations per month was compared across the four seasons—both for all patients and within separate gender and ten-year age groups—using generalized linear models with Poisson distribution, identity link function and Bonferroni multiple comparisons. The boundaries of each season followed the meteorological definition for the South Hemisphere [15], i.e. winter is comprised of June, July and August; spring is comprised of September, October and November; summer includes December, January and February, and autumn includes March, April and May. Monthly average temperature levels (mean ± standard deviation) were compared across the four seasons by analysis of variances and Bonferroni multiple comparisons. The Box-Jenkins autoregressive integrated moving average (ARIMA) model was used to test the association between ambient temperature and the average number of hospitalization per month. [16] The modeling process proceeded in three steps: first, the temporal effect of the series of cardiovascular events was removed by the ARIMA model; second, model parameters were chosen according to the autocorrelations and partial autocorrelations; and finally, the white noise or stationary time series was used to estimate the coefficients. For each series in the study, a regression parameter was added to test the influence of model specifications on the significance of obtained results. A p<0.05 was considered statistically significant. All statistical analyses were performed using SPSS statistical software version 20.0.

## Results

**Fig 1:**
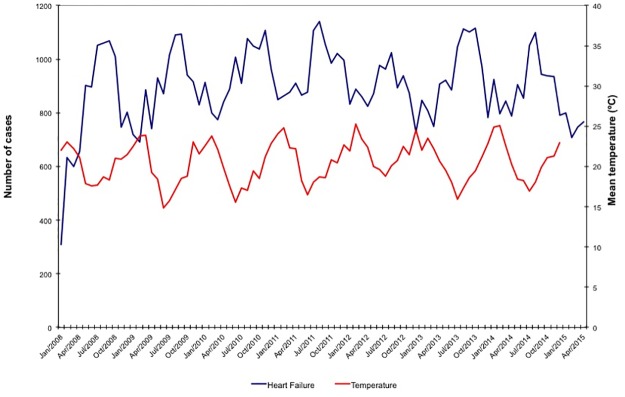
Monthly average temperature levels and number of hospitalizations per month for heart failure.

**Fig 2:**
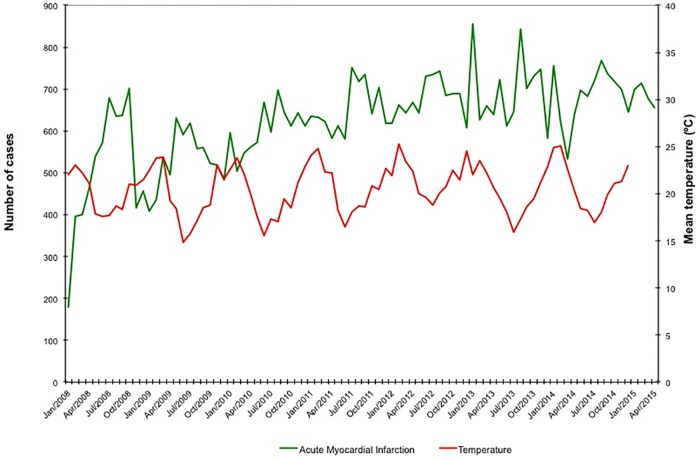
Monthly average temperature levels and number of hospitalizations per month for acute myocardial infarction.

During the study period, the National Registry of Public Health recorded a total of 4,823,761 hospital admissions. Using the inclusion criteria outlined above, we identified 76,474 hospitalizations for HF (49.9% men, 67.7% ≥ 60 years) and 54,561 hospitalizations for AMI (63.3% men, 55.9% ≥ 60 years). Monthly average temperature levels and the number of hospitalizations for HF and AMI per month are illustrated in Figs 1 and 2. Seasonal average temperature levels were lowest in the winter (mean ± SD of 17.5 ± 1.3°C), moderately lower than the monthly average temperature recorded during the spring, summer and autumn (respectively, mean ± SD of 20.3 ± 1.4, 23.3 ± 1.2 and 20.7 ± 1.9; p <0.001 for comparison of winter vs. all other seasons).

The average number of hospital admissions for HF and AMI per month peaked during winter, with an increase of up to 30% for HF and 16% for AMI, when compared to summer, which was the season with lowest figures for both diseases (respectively, HF: 996 *vs*. 767 per month, p<0.001; and AMI: 678 *vs*. 586 per month, p<0.001). These findings were not affected by gender (Tables 1 and 2). When analyzed within age groups, the average number of admissions for HF per month during winter was higher in patients older than 40, while the equivalent figure for AMI was higher in patients older than 50 (Tables 1 and 2). Using ARIMA analysis, we find that the average number of hospitalizations for AMI and HF per month increases during winter months; however, we did not find an association between temperature levels and hospitalizations (Table 3).

**Table 1:** Average number of hospitalizations per month for heart failure, per season, according to gender and age group.

Characteristics of pts. with Heart Failure	Hospitalizations per Month					p
	Jan-Dec	Winter	Spring	Summer	Autumn	
**Gender**						
Female	435	494	456	381	414	**<0.001**
Male	434	502	451	386	406	**<0.001**
**Age Group (years)**						
20 to 29	14	15	13	14	14	0,102
30 to 39	33	34	34	31	33	0,108
40 to 49	75	85	73	70	73	**<0.001**
50 to 59	159	181	164	141	152	**<0.001**
60 to 69	206	233	216	184	194	**<0.001**
70 to 79	211	244	225	183	196	**<0.001**
80+	171	203	182	144	158	**<0.001**
**Total**	**869**	**996**	**907**	**767**	**820**	<0.001*

*P<0.001 for comparisons between each season vs. the other three.

**Table 2:** Average number of hospitalizations per month for acute myocardial infarction, per season, according to gender and age group.

Characteristics of pts. with AMI	Hospitalizations per Month					p
	Jan-Dec	Winter	Spring	Summer	Autumn	
**Gender**						
Female	228	250	231	209	223	**<0.001**
Male	392	429	394	378	372	**<0.001**
**Age Group (years)**						
20 to 29	4	4	3	4	4	0,116
30 to 39	18	18	18	19	17	0,685
40 to 49	79	81	81	77	78	0,298
50 to 59	172	185	172	170	164	**<0.001**
60 to 69	171	187	176	159	164	**<0.001**
70 to 79	117	135	118	106	110	**<0.001**
80+	59	67	57	52	58a	**<0.001**
**Total**	**620**	**678**	**626**	**586**	**595**	<0.001*

*P<0.001 for comparisons between winter vs. other seasons, and spring vs. other seasons. There was no difference between summer and autumn. AMI, acute myocardial infarction. AMI, acute myocardial infarction

**Table 3:** ARIMA analysis on the seasonal variation of the rates of hospitalizations for acute myocardial infarction and heart failure, and on the association between hospitalization rates and temperature levels.

	Parameter	coefficient	Std. Error	T value	p
**Heart Failure**	MA*1.12	-0.29318	0.11708	-2.5	0.012
	Temperature	0.10976	0.32371	0.34	0.734
**Acute Myocardial Infarction**	MA*1.12	-0.35871	0.11726	-3.06	0.002
	Temperature	0.1978	0.33522	0.59	0.555

*MA = moving average.

## Discussion

In our analysis of data for Sao Paulo, we find an increase in the average number of hospitalizations per month for HF and AMI during the winter when compared to each of the other seasons, and an increase of up to 30 and 16%, respectively, when winter figures are compared to summer. The monthly average temperature during winter was only moderately lower than in other seasons, and we did not observe an association between ambient temperature and hospital admissions.

Numerous studies examining data from high-income countries with temperate climates have demonstrated an association between winter and the incidence and mortality from HF and AMI. [17–19] In these countries, the oscillation in temperature levels across seasons is quite large, with a marked decrease during the winter. Extremely cold temperatures can induce systemic adrenergic activation, increased peripheral vascular resistance, and systemic hypertension resulting in increases in both cardiac oxygen consumption and demands on cardiac output.[20, 21] Low ambient temperatures can also induce the release of fibrinogen and coagulation factors that may result on a hypercoagulable state and a higher risk of atherothrombosis. [22]

Similar assessments on the effects of winter on cardiovascular risk in low- and middle-income tropical countries are scarcer but still suggestive. A study conducted in Havana (Cuba), found higher mortality in AMI patients during winter.[8] In Bangladesh, a study using one small registry found that hospital admission rates for several cardiovascular decompensated diseases, including AMI and HF, peaked during the winter.[9] Some recent studies have shown that this increase in cardiovascular risk can be attributed, at least partially, to a decline in temperature. One large multinational study that tested the association between ambient temperature and general mortality found that most of the temperature-related deaths could be attributed to low temperatures typical of winter and not to high temperatures typical of summer. [23] Even if this association was more evident in high-income temperate countries, it was still present in low- and middle-income tropical countries such as Brazil and Thailand. Studies conducted in Taipei and Hong Kong also found that low temperature days and daily temperature oscillation are associated with increased risk for AMI and HF.[24–26] Similar to Sao Paulo, Taipei and Hong Kong have a humid subtropical climate and mild winters. Unlike Sao Paulo, however, they have a slightly wider gap in monthly average temperatures across seasons [27] and a much higher income per capita. [28] These differences hinder a direct comparison across these sites but they also suggest that lower temperatures might not be the only variable affecting the observed association between winter and increased hospitalizations for HF and AMI.

Two previous studies have examined the influence of winter on cardiovascular risk in Sao Paulo, Brazil. One study found an increase in mortality for AMI during winter but it relied exclusively on data for a single year (1997) [29]. Another study examined a larger dataset and found that hospitalization for HF increases during winter. [30] However, this study did not test whether this perceived increase was statistically significant and neither did it incorporate ambient temperature into its analysis.

In our study, we found an increase in hospitalizations for HF and AMI during the winter despite a moderate average winter temperature of 17.5°C, only 2.8 to 5.8°C lower than the other seasons. Besides the direct effects of low ambient temperatures on the cardiovascular system, environmental aspects linked to winter could help explain our findings. Sao Paulo winters are characterized by low humidity, limited rains and a higher frequency of thermal inversions, which occur when cold air gets trapped near the surface and underneath a layer of warmer air. [31] Together, these conditions prevent the dispersion of pollutants such as carbon monoxide (CO), nitrogen dioxide (NO2), sulfur dioxide (SO2), and inhalable particulate matter (PM10) that are associated with increased cardiovascular risk. [32] Lower ambient temperatures, low humidity, and high pollution could also reinforce each other as they contribute to a higher incidence of respiratory diseases and influenza, with a consequent increase in cardiovascular risk.[33]

On the socio-economic side, Sao Paulo is the economic engine of Brazil, with an estimated 11,253,503 inhabitants. [34] The city has been characterized by several decades of mostly unplanned urban growth. At present, approximately 40% of the population of its larger metropolitan area live in precarious housing conditions.[35] Moreover, its inhabitants are often exposed to massive traffic jams and significant pollution. [36] These issues are especially salient to its low-income citizens, a group that relies heavily on the services provided by the Brazilian Public Health System. The importance of socio-economic variables on winter risk has been noted in a recent multicenter study conducted in 14 European countries. This study found that Portugal had the highest rate of excess winter mortality, even though it has a milder winter than several other countries included in the analysis. [37] This study also demonstrated that per capita national income, per capita health expenditure, poverty rates, and several indicators of residential thermal standards were associated with excess winter mortality. These findings support the hypothesis that not only temperature levels, but also adverse socio-economic conditions may increase the effect of winter on cardiovascular risk.

Our study has several limitations. First, this was an observational study and despite statistical adjustments, a causal and definite relationship between seasonality and hospital admissions for HF and AMI cannot be determined. Second, we analyzed data maintained by the Brazilian Public Health System that accounts for 66% of hospital admissions in the country [38]. Data pertaining to the private health system were not available. Third, our dataset does not contain information on clinical characteristics besides age and sex. Similarly, we lacked data on pollution levels. For these reasons, we could neither adjust our results for comorbidities nor explore the direct effect of pollution on cardiovascular risk during winter. Finally, the Brazilian National Registry of Public Health provides only monthly data on hospital admissions so we could not analyze daily or weekly variation that could have revealed an association between cold days and an increase in cardiovascular risk. In spite of these limitations, our analysis of data for Sao Paulo demonstrates the seasonality of hospital admissions for HF and AMI, with an increase during relatively mild winters. Considering that mean temperatures were only moderately lower during the winter when compared to other seasons, our study raises the possibility that other factors related to disorderly urban occupation and urban poverty, such as a greater exposure to pollution and the lack of thermal protection in precarious housing, might also be associated with the seasonal increase in cardiovascular risk. This broader issue deserves further investigation.

In conclusion, in the megacity of Sao Paulo, winters were associated with an increased number of hospitalization for both HF and AMI. This relationship did not hinge on seasonal temperature oscillations. Our findings suggest that the prevention of cardiovascular disease decompensation should be emphasized during winter even in low to middle-income countries with modest temperature oscillations.

## Supporting information

S1 Dataset For hospital admissions for heart failure and acute myocardial infarction. (XLS)

## Abbreviations

AMI: acute myocardial infarction
ARIMA: autoregressive integrated moving average
HF: heart failure
